# Is there an association of blood cadmium level with nonmelanoma skin cancer: results from a cross-sectional study

**DOI:** 10.3389/fpubh.2024.1507492

**Published:** 2025-01-10

**Authors:** Dan Li, Jigang Chen, Rui Feng, Yanni Wang

**Affiliations:** Department of Burn and Plastic Surgery, Beijing Children’s Hospital, Capital Medical University, National Center for Children's Health, Beijing, China

**Keywords:** cadmium, nonmelanoma skin cancer, restricted cubic splines, National Health and Nutrition Examination Survey, cancer

## Abstract

**Objective:**

Nonmelanoma skin cancer (NMSC) is a common malignancy that starts in the top layer of the skin. Exposure to heavy metals has been suggested to increase the risk of skin cancer. Cadmium, prevalent in various industries and present in cigarette smoke, has been implicated in potential skin effects in animal studies. However, the impact of chronic cadmium exposure on NMSC development remains largely unexplored. This study aimed to investigate the relationship between blood cadmium levels and the odds of NMSC.

**Methods:**

We analyzed cross-sectional data from the National Health and Nutrition Examination Survey, covering the years 1999–2000 to 2017–2018. NMSC cases were identified through self-reported physician diagnoses. We assessed the association between blood cadmium levels—considered as both a continuous variable and in quartiles—and the odds of NMSC using multivariable logistic regression models. Restricted cubic splines (RCS) were incorporated to estimate the non-linear relationship between cadmium levels and NMSC.

**Results:**

The study included 41,577 participants, with 952 reporting NMSC and 40,625 without any cancers. No association was found between cadmium levels and NMSC when analyzed as either a continuous or quartile variable. However, RCS revealed an inverted U-shaped curve indicating a relationship between blood cadmium levels and NMSC odds. This pattern persisted when focusing exclusively on non-Hispanic White participants.

**Conclusion:**

A statistically significant relationship was observed between blood cadmium levels and the odds of NMSC, though the increased odds were only evident at low cadmium levels. Further research is necessary to investigate the causality and underlying mechanisms involved.

## Highlights

- The association of blood cadmium level with the odds of nonmelanoma skin cancer (NMSC) was evaluated for the first time.
- This study has some strengths including the reliability of the NHANES data as well as its large sample size.
- The diagnosis of NMSC was based on self-report and it was not confirmed by medical or pathological reports.
- NHANES was a cross-sectional design and the casual inference between cadmium and NMSC should not be made.

## Introduction

Nonmelanoma skin cancer (NMSC) is the most common malignancy that starts in the top layer of skin, encompassing basal cell carcinoma, squamous cell carcinoma, and other rarer skin tumors (1). The incidence of NMSC is rising globally due to the aging population (2). According to the World Health Organization, approximately 2 to 3 million cases of NMSC occur worldwide each year (3). Due to its high prevalence, the financial burden of NMSC treatment is significant for both patients and society. In the United States, the average annual cost of treating NMSC is estimated at $4.8 billion (4). The cause of NMSC is not fully understood. Identifying the risk factors is crucial for the prevention and early detection of NMSC to reduce its burden.

Exposure to heavy metals has been suggested to increase the risk of skin cancer (5).

Cadmium is an element found in low concentrations in the Earth’s crust. Several environmental sources contribute to cadmium pollution, including fossil fuels, nonferrous industries, fertilizers, and sewage sludge (6). The general population is exposed to cadmium through contaminated water and food, with cigarettes representing another significant source of exposure (7). Cadmium is highly toxic and is classified as a Group I carcinogen by the International Agency for Research on Cancer. It has been associated with tumors in various parts of the human body, including the lungs, liver, kidneys, blood, and prostate (8).

*In vitro* study have indicated a potential interaction between cadmium and keratinocytes (9, 10). Additionally, cadmium has been shown to act as a co-mutagen with ultraviolet radiation, which can lead to damage in both keratinocytes and melanocytes (11). These findings suggest that cadmium may be a risk factor for the development of NMSC. However, to our knowledge, no epidemiologic studies have investigated the association between cadmium exposure and NMSC. Preventive measures regarding cadmium pollution could be more effectively implemented if a dose–response relationship were established between cadmium levels and the risk of NMSC. The complex association between cadmium and NMSC warrants further exploration using various statistical methods to verify if there were significant differences in cadmium levels between individuals with NMSC and those without cancer. Therefore, we utilized publicly available data from the National Health and Nutrition Examination Survey (NHANES) to examine whether blood cadmium levels are related to the odds of NMSC.

## Methods

### Study population

This study was conducted using cross-sectional data from the NHANES spanning from 1999–2000 to 2017–2018. The NHANES data is publicly available online (12). In brief, NHANES is an ongoing complex sample survey that includes in-home interviews, physical examinations, and laboratory tests. It collects information on the demographics, health, and nutritional status of the civilian noninstitutionalized population in the United States. Since 1999, data has been continuously collected and released in 2-year cycles. The study protocols for NHANES were approved by the National Center for Health Statistics Institutional Review Board, and written informed consent was obtained from each participant (Approval ID: Protocol #98-12; Protocol #2005-06; Protocol #2011-17; Protocol #2018-01) (13). Our participants were limited to individuals aged 20 years or older, and those without information on blood cadmium levels or cancer diagnoses were excluded.

### Exposure and outcome measurements

The exposure of interest is whole-blood cadmium level. During physical examinations, blood samples were collected from participants. The samples were processed and sent to a central laboratory for testing. A detailed description of the laboratory methodology for whole-blood cadmium measurement can be found on the NHANES website. In brief, whole-blood cadmium concentration was determined using inductively coupled plasma mass spectrometry (ICP-MS), a multi-element analytical technique based on quadrupole ICP-MS technology. For values below the detection limit, an imputed fill value was placed in the analyte results field. This value is the lower limit of detection divided by the square root of 2.

The outcome was based on self-reported data collected from the medical condition questionnaire during personal interviews. If participants answered “yes” to the question, “Have you ever been told by a doctor or other health professional that you had cancer or a malignancy of any kind?”, they were then asked, “What kind of cancer?”. We created a binary variable for participants with NMSC and those without any cancers.

### Covariates

Important covariates identified in the literature on NMSC included age, sex, race/ethnicity, education, income, body mass index (BMI), hypertension, diabetes, dyslipidemia, current smoking status, alcohol use, hours of physical activity per week, use of sunscreen or protective clothing, human papillomavirus (HPV) infection, and urinary arsenic level. Race/ethnicity was categorized into four groups: non-Hispanic white, non-Hispanic black, Mexican American, and others. Education levels included less than high school, high school graduate, some college, and college graduate or higher. Income was assessed using the income-to-poverty ratio, defined as annual family income divided by the poverty threshold, adjusted for inflation and family size. BMI was calculated as weight (in kilograms) divided by height (in meters). Hypertension was defined as a systolic blood pressure of 130 mm Hg or higher, diastolic blood pressure of 80 mm Hg or higher, or the use of antihypertensive medications. Diabetes was defined as a hemoglobin A1c level of 6.5% or higher, or the use of antidiabetic medications. Dyslipidemia was defined as a total cholesterol level of 240 mg/dL or higher, or the use of lipid-lowering medications. Current smoking status was evaluated based on participants’ responses about whether they were currently smoking. Alcohol use was classified into three categories: equal to or less than 1 drink per week, between 1 to 5 drinks per week, and more than 5 drinks per week, with a “drink” defined as a 12 oz. beer, a 5 oz. glass of wine, or 1.5 ounces of liquor. Physical activity was calculated as hours of moderate-intensity activity plus twice the minutes of vigorous-intensity activity (14). The use of sunscreen or protective clothing was assessed based on participants’ responses about using sunscreen or wearing long-sleeved shirts. Vaginal swab samples were collected from female participants, and HPV infection was detected using the Roche Linear Array HPV Genotyping test based on HPV L1 consensus polymerase chain reaction. Participants were asked to provide spot urine samples, and total urinary arsenic levels were determined by inductively coupled plasma dynamic reaction cell mass spectrometry.

### Statistical analysis

All statistical analyses were conducted in R version 4.1.3 using the “Survey” package. The complex sampling design, which included the primary sampling unit, strata, and weights, was considered during the analysis. Following NHANES recommendations, appropriate 20-year sampling weights were constructed for subsamples to ensure accurate estimation of associations and variance (12). All statistical tests were two-sided, and a *p*-value of less than 0.05 was considered statistically significant.

Continuous variables were presented as means, while categorical variables were presented as percentages. The levels of cadmium and arsenic were right-skewed and were natural log-transformed for the analysis. The mean level of cadmium before transformation was 0.054 mg/dL and after transformation was 0.38 mg/dL; the mean level of arsenic before transformation was 19.6 μg/L and after transformation was 8.4 μg/L. The differences in continuous variables between those with NMSC and those without cancer were examined using a t-test, while differences in categorical variables were assessed using a chi-squared test. Binomial logistic regression analysis was applied to investigate the association between cadmium levels and the odds of NMSC. After natural log-transformation, cadmium levels were first modeled as a continuous variable in the regression analysis. Second, they were divided into quartiles and modeled as an ordinal variable, with the first quartile serving as the reference. To identify a potential non-linear relationship between cadmium levels and the odds of NMSC, restricted cubic splines (RCS) were employed in the regression analysis. RCS offers the advantage of parsimony and allows for smooth dose–response curves with a wide range of possible shapes, reflecting the relationship between continuous exposure and an outcome (15). The number of knots in the RCS was determined based on the smallest Akaike’s Information Criterion, with three knots applied according to the results. The *p*-value for the non-linear trend was assessed using the Wald test for the coefficient of RCS. Additionally, sensitivity analyses were conducted to test whether the association between cadmium and NMSC varied across different subgroups defined by age (20–44 years, 45–64 years, over 65 years), sex (male and female), race, education, and income (income-to-poverty ratio < 1.3 and ≥ 1.3). A two-way interaction term between the quartile of cadmium level and subgroup status was added to the regression model. Further stratified analyses would be conducted if a difference was detected in a specific subgroup.

We developed 3 models for the regression analysis. No covariables were adjusted in model 1, and age, sex, race/ethnicity, education, and income were adjusted in model 2. In model 3, BMI, hypertension, diabetes, dyslipidemia, current smoke, alcohol use, hours of physical activity, sunscreen or protective clothing, HPV infection, and urinary arsenic level were further adjusted based on model 2.

## Results

A total of 112,339 participants were included in NHANES from 1999–2000 to 2017–2018. Among them, 55,513 were adults aged 20 years or older. Of these participants, 13,936 were excluded due to missing information on cadmium levels or cancer diagnoses, resulting in a total of 41,577 subjects available for analysis. Of these, 952 reported having NMSC, while 40,625 did not have any cancers.

The differences between participants with NMSC and those without any cancers are reported in Table 1. Compared to individuals without any cancers, those with NMSC were older and more likely to be men and non-Hispanic White. They had lower levels of education and income and were more likely to have hypertension, diabetes, and dyslipidemia. Additionally, they were less likely to engage in current smoking or physical activity. Furthermore, participants with NMSC were more likely to use sunscreen or wear protective clothing compared to their controls.

**Table 1:** Characteristics of participants with and without NMSC.

Characteristics	With NMSC	Without any cancers	*p*-value
Total, *n*	952	40,625	
Mean age, year	63.4 (0.6)	45.2 (0.2)	<0.001
Men, %	53.3 (2.1)	48.7 (0.3)	0.038
Non-Hispanic White, %	97.5 (0.5)	67.0 (1.1)	<0.001
Less than high school education, %	10.6 (1.1)	18.1 (0.5)	<0.001
Income-to-poverty ratio < 1.3, %	7.7 (1.1)	20.5 (0.5)	<0.001
BMI	28.4 (0.3)	28.6 (0.1)	0.476
Hypertension, %	68.1 (2.0)	44.9 (0.5)	<0.001
Diabetes, %	13.3 (1.3)	9.3 (0.2)	<0.001
Dyslipidemia, %	40.6 (2.2)	25.6 (0.4)	<0.001
Current smoke, %	11.5 (1.4)	22.2 (0.4)	<0.001
Alcohol use, %			0.227
<= 1 drinks per week	53.8 (2.8)	56.6 (0.8)	
>1, <=5 drinks per week	20.3 (2.0)	21.2 (0.5)	
> 5 drinks per week	25.9 (2.3)	22.1 (0.5)	
Hours of physical activity per week	10.1 (0.8)	16.4 (0.3)	<0.001
Sunscreen or protective clothing, %	76.9 (4.6)	62.7 (0.6)	0.008
HPV infection	73.8 (6.2)	58.4 (0.8)	0.032
Total urinary arsenic, ug/L	8.6 (0.11)	7.4 (0.02)	0.192
Cadmium, ug/L	0.37 (0.02)	0.36 (0.01)	0.415

All the data were presented as mean or percentage with standard error in parenthesis. *The level of cadmium and urinary arsenic was presented as geometric mean. NMSC: nonmelanoma skin cancer; BMI: body mass index.

**Figure 1:**
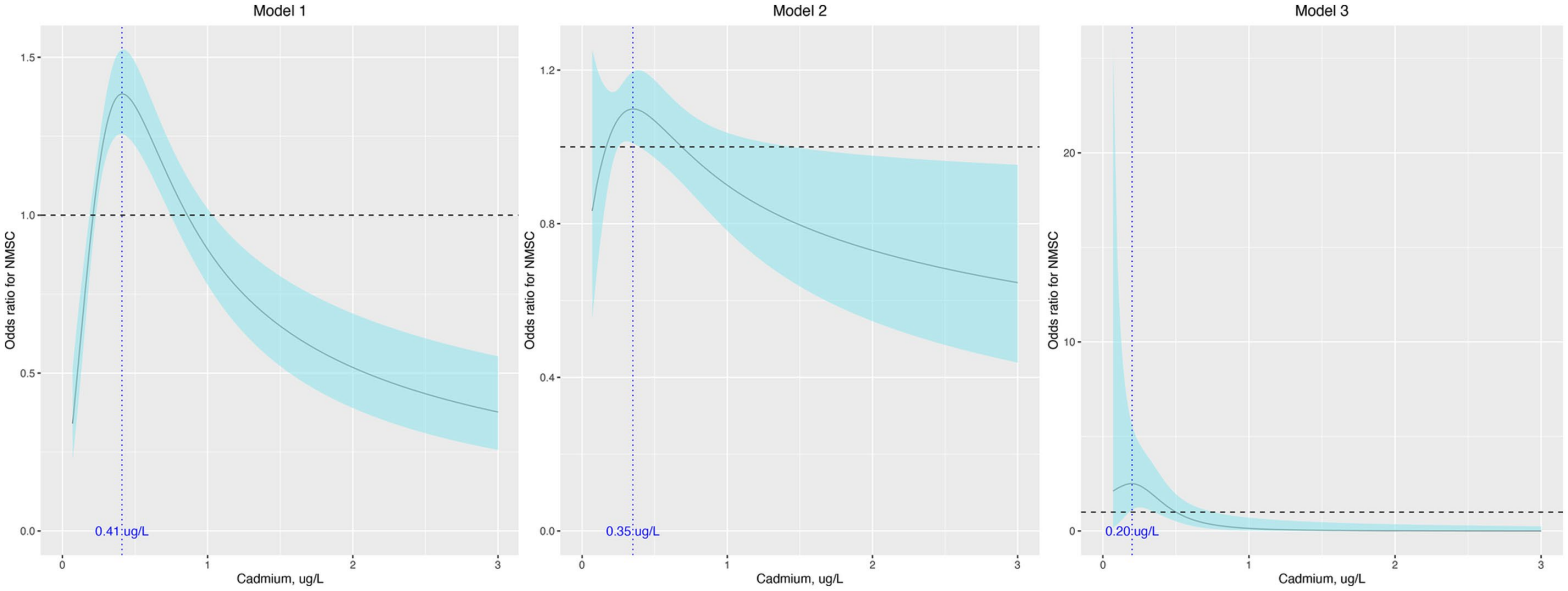
Odds ratio (95% confidence interval) of NMSC by blood cadmium level. The odds ratio (the solid lines) and its 95% confidence interval (the curved lines) were determined based on logistic regression analysis with restricted cubic splines for natural log-transformed cadmium level. No covariates were adjusted in model 1. Age, sex, race, education, and income were adjusted in model 2. Age, sex, race, education, income, body mass index, hypertension, diabetes, dyslipidemia, current smoke, alcohol use, hours of physical activity, sunscreen or protective clothing, human papillomavirus infection, and urinary arsenic level were adjusted in model 3. NMSC: nonmelanoma skin cancer.

When treated as a continuous variable, the association between cadmium levels and the odds of NMSC was insignificant in model 1 (odds ratio [OR], 1.04; 95% confidence interval [95% CI], 0.95–1.14), model 2 (OR, 0.944; 95% CI, 0.83–1.08), and model 3 (OR, 0.36; 95% CI, 0.11–1.18). When analyzed as quartiles, the association was significant only in model 1 (*p* for trend, 0.011), with participants in the second and third quartiles showing higher odds of NMSC compared to those in the first quartile (Table 2). The results of the regression analysis using restricted cubic splines (RCS) are presented in Figure 1. We found that cadmium was significantly associated with NMSC in an inverted U-shaped manner across model 1, model 2, and model 3 (all *p*-values for non-linearity were less than 0.05).

**Table 2:** Odds ratio (95% confidence interval) of NMSC associated with quartiles of cadmium levels.

	Quartile 1 (≤0.20 ug/L)	Quartile 2 (0.20–0.33 ug/L)	Quartile 3 (0.33–0.60 ug/L)	Quartile 4 (>0.60 ug/L)	*P* for trend
NMSC (*n*)	152	215	390	195	
Model 1	Reference	1.89 (1.46–2.44)	2.13 (1.65–2.74)	1.26(0.96–1.66)	0.011
Model 2	Reference	1.27 (0.96–1.68)	1.17 (0.89–1.55)	0.99 (0.74–1.33)	0.849
Model 3	Reference	0.82 (0.50–3.14)	1.26 (0.65–3.50)	1.1*10^−8^ (9.6*10^−10^-1.4*10^−7^)	0.282

NMSC: nonmelanoma skin cancer. No covariates were adjusted in model 1. Age, sex, race, education, and income were adjusted in model 2. Age, sex, race, education, income, body mass index, hypertension, diabetes, dyslipidemia, current smoke, alcohol use, hours of physical activity, sunscreen or protective clothing, human papillomavirus infection, and urinary arsenic level were adjusted in model 3.

**Figure 2:**
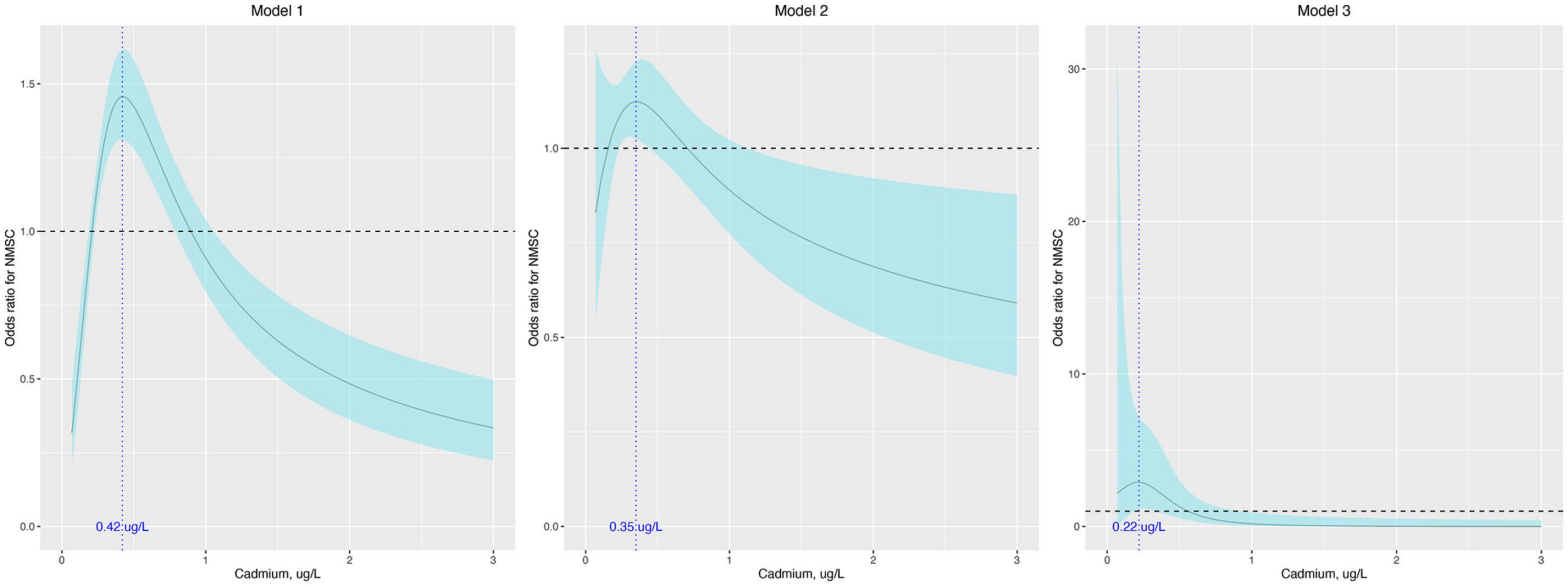
Odds ratio (95% confidence interval) of NMSC by blood cadmium level when limiting the participants to non-Hispanic White. The odds ratio (the solid lines) and its 95% confidence interval (the curved lines) were determined based on logistic regression analysis with restricted cubic splines for natural log-transformed cadmium level. No covariates were adjusted in model 1. Age, sex, race, education, and income were adjusted in model 2. Age, sex, race, education, income, body mass index, hypertension, diabetes, dyslipidemia, current smoke, alcohol use, hours of physical activity, sunscreen or protective clothing, human papillomavirus infection, and urinary arsenic level were adjusted in model 3. NMSC: nonmelanoma skin cancer.

In the sensitivity analysis, the association between cadmium and NMSC was generally consistent across different subgroups, except for race (Table 3). According to our results, the majority of patients with NMSC were non-Hispanic White (97.5%). When limiting our analysis to non-Hispanic White participants, RCS indicated that cadmium remained associated with NMSC in a similar non-linear manner across all three models (Figure 2).

**Table 3:** *p* values for the interaction of cadmium levels by quartiles with different subgroups.

	Age	Sex	Race	Education	Income
Model 1	0.919	0.831	**<0.001**	0.694	0.626
Model 2	0.779	0.346	**0.043**	0.353	0.556
Model 3	0.953	0.069	0.231	0.189	0.594

No covariates were adjusted in model 1. Age, sex, race, education, and income were adjusted in model 2. Age, sex, race, education, income, body mass index, hypertension, diabetes, dyslipidemia, current smoke, alcohol use, hours of physical activity, sunscreen or protective clothing, human papillomavirus infection, and urinary arsenic level were adjusted in model 3. Bold values meant statistically significant.

## Discussion

As a toxic heavy metal, cadmium has significant adverse effects on both occupational and environmental health (16). As a cumulative toxin, cadmium has an extremely long biological half-life (16). It has been recognized as a human carcinogen by the World Health Organization’s International Agency for Research on Cancer and the US National Toxicology Program (17, 18). According to most research, occupational exposure to cadmium increases the risk of lung cancer, making the lung the most established site for cadmium-induced cancer (19). The recognition of cadmium as a human carcinogen by regulatory agencies is largely due to the established relationship between occupational exposure and lung cancer (17, 18). Additionally, cadmium has been linked to cancers of the liver, prostate, kidney, bladder, hematopoietic system, and stomach in humans (20). Nevertheless, the link between cadmium exposure and cancers at skins requires further epidemiological and experimental investigation.

Based on this large sample size, general population-based cohort study, we investigated the relationship between blood cadmium levels and odds of NMSC. While cadmium was not associated with NMSC when treated as a continuous or quartile variable, we identified a significant non-linear relationship between them even after adjusting for potential covariates. The U-shaped dose-effect curve indicated that moderately elevated levels of blood cadmium were associated with increased odds of NMSC, while significantly elevated levels were inversely associated with that odds. This unexpected result is difficult to explain, especially given that a positive non-linear association between dietary cadmium intake and the risk of melanoma has been observed in population studies (21). However, considering the complex metabolism of cadmium in human organs (22, 23), the concentration of cadmium in the diet does not necessarily equal its concentration in the blood, nor does blood concentration directly reflect levels in the skin. Moreover, our finding is not unprecedented; an inverse association between blood cadmium levels and the risk of breast cancer has also been indicated in the European Prospective Investigation into Cancer and Nutrition-Italy cohort and in a meta-analysis (24, 25). Further studies are needed to explore the underlying mechanisms of the inverse relationship between blood cadmium and the risk of NMSC.

To our knowledge, there are no human studies that have investigated the relationship between cadmium and NMSC. However, cadmium has been identified as a co-mutagen with ultraviolet radiation, which can lead to damage of keratinocytes and melanocytes (11). Additionally, results from experimental studies suggest a potential correlation between cadmium and NMSC. In an *in vitro* study, Wester et al. demonstrated that human skin absorbs more cadmium from contaminated soil and water than from plasma (26). Later, Lansdown et al. applied a cadmium chloride solution to the shaved skin of rats and mice for 10 days, observing dose-related damages such as dermal hyperkeratosis and acanthosis, along with ulcerative changes and an increased mitotic index of skin cells (27). These animal studies strongly suggest that cadmium can be absorbed through the skin, though the actual mechanisms require further investigation. It may bind to sulfhydryl radicals of cysteine in epidermal keratins or induce complexing with metallothionein (28). Together, these studies indicate that cadmium might be a potent carcinogen for the skin.

Only a few studies have investigated the potential carcinogenic effects of cadmium exposure on human skin. Kim et al. performed a case–control study with 138 individuals diagnosed with skin cancers of all types and 142 controls. They found that the level of blood cadmium was not different among two groups (29). In another study, Weinlich et al. enrolled 760 patients with primary cutaneous melanoma in a prospective cohort study and found that increased levels of metallothionein—a protein to which cadmium has a strong affinity—were linked to a higher risk of disease progression (30). In Italy, Vinceti et al. studied 58 participants with cutaneous melanoma and 58 controls, measuring the concentration of cadmium in the toenails of both groups. However, they found no relationship between toenail cadmium levels and melanoma (31). In another study conducted by Vinceti et al., the researchers evaluated cadmium intake among 380 participants with melanoma and 719 controls, finding a positive non-linear association between cadmium intake and the odds of melanoma (21). Together, these studies suggest a possible, albeit not very strong, link between cadmium exposure and the risk of melanoma.

The limitations of this study should be acknowledged. First, the diagnosis of NMSC was based on self-report and was not confirmed by clinical or pathological records, which may have led to some outcome misclassification. Second, only one routine blood test was conducted to measure blood cadmium levels; serial blood tests might provide more reliable information. Third, since NHANES employs a cross-sectional design, causal inferences between cadmium and NMSC should not be drawn. Additionally, there may be a degree of recall or selection bias, as much of the NHANES data is based on self-reported personal interviews.

## Conclusion

Our study demonstrated a statistically significant association between blood cadmium levels and odds of NMSC. However, the increased risk was observed only within the range of low cadmium levels. Further prospective studies are warranted to determine causality and the underlying mechanisms.

## Data Availability

The original contributions presented in the study are included in the article/supplementary material, further inquiries can be directed to the corresponding author.
